# Evidence of Rat Hepatitis E Virus Circulation through Wastewater Surveillance, Central Argentina

**DOI:** 10.3201/eid3205.260304

**Published:** 2026-05

**Authors:** Florence Abravanel, Clément Castille, Nicolas Marter, Jean Luc Guerin, Sébastien Lhomme, Jacques Izopet

**Affiliations:** CHU Toulouse Purpan, INSERM UMR1291, CNRS UMR5051, Université de Toulouse, Toulouse, France (F. Abravanel, S. Lhomme, J. Izopet); ENVT, Université de Toulouse, INRAE, IHAP, Toulouse (C. Castille, J.L. Guerin); Eau de Toulouse Métropole, Toulouse (N. Marter).

**Keywords:** Rat hepatitis E virus, R-HEV, *Rocahepevirus ratti*, wastewater-based epidemiology, viruses, zoonoses, enteric infections, Argentina

**To the Editor:** A recently published dispatch about rat hepatitis E virus (rHEV) detected high levels of rHEV in wastewater samples from Argentina (67.7%) (*1*). The study authors claimed their findings supported further investigation of the virus in animal reservoirs and humans, with a focus on hepatitis cases of unknown etiology.

rHEV is genetically distinct from conventional human-infecting hepatitis E virus (HEV; *Paslahepevirus balayani*) and is not detected by the PCRs that detect HEV RNA. A recent study in Spain detected an rHEV frequency of 1.4% among patients with hepatitis of unknown etiology (*2*). The rHEV spillover mechanism to humans is unclear.

We analyzed rHEV in wastewater samples from Toulouse, France, where HEV is endemic (HEV IgG seroprevalence 47.8%) (*3*). We collected 49 wastewater samples weekly during 2025 and detected rHEV in all by using a previously published protocol (*4*). However, we could not sequence the genomes because of low viral concentration. During 2023–2025, we tested 484 immunocompetent patients with a positive HEV IgM result by using Liaison (Diasorin, https://us.diasorin.com), to detect HEV IgM in patients with rHEV infection (*5*), and 578 immunocompromised patients living in the same area. We collected the samples at infection onset when the liver enzymes were elevated and AltoStar HEV PCR Kit (Altona Diagnostics, https://altona-diagnostics.com) results were negative. None of the samples were positive for rHEV RNA.

The high detection rate of rHEV in wastewater reflecting high circulation in urban rodents contrasts with the rarity of human cases. This contrast could be linked to low exposure of humans to contaminated sources, low human infection capability of rHEV, or cross protection because of immunity conferred by HEV in Toulouse, where the seroprevalence is higher than in Spain, where rHEV human cases are more frequent (*2*). Future studies to evaluate rHEV-specific serologic response could be useful.
